# Supplementary material for: SLFN11 counteracts the RFWD3-PRIMPOL DNA damage tolerance axis to restrain gapped DNA synthesis in response to replication stress
Source: Nat Commun. 2025 Dec 10;16:11029. doi: 10.1038/s41467-025-66068-1 (PMC12696100; doi:10.1038/s41467-025-66068-1)
Supplement: Supplementary file 1 — Supplementary Information [file 41467_2025_66068_MOESM1_ESM.pdf]

Supplementary Information

SLFN11 counteracts the RFWD3-PRIMPOL DNA damage tolerance axis to restrain gapped DNA synthesis in response to replication stress

Kate E. Coleman[1]#, Dong-Woo Shin[2,3]#, Liana Goehring[1]#, Beata Szeitz[1,4], David Fenyö[1,4], Eli Rothenberg[1], John T. Poirier[2,3]* and Tony T. Huang[1]*

[1]Department of Biochemistry & Molecular Pharmacology, New York University Grossman School of Medicine, New York, NY, USA
[2]Department of Medicine, Laura and Isaac Perlmutter Cancer Center, New York University Grossman School of Medicine, New York, NY, USA
[3]Laura and Isaac Perlmutter Cancer Center, New York University Langone Health, New York, NY, USA
[4]Institute for Systems Genetics, New York University Grossman School of Medicine, New York, NY, USA

[#]These authors contributed equally to this study.
*Corresponding authors: Tony.huang@nyulangone.org; John.Poirier@nyulangone.org

This File includes:

Supplementary Figures 1-7

Supplementary Table 1

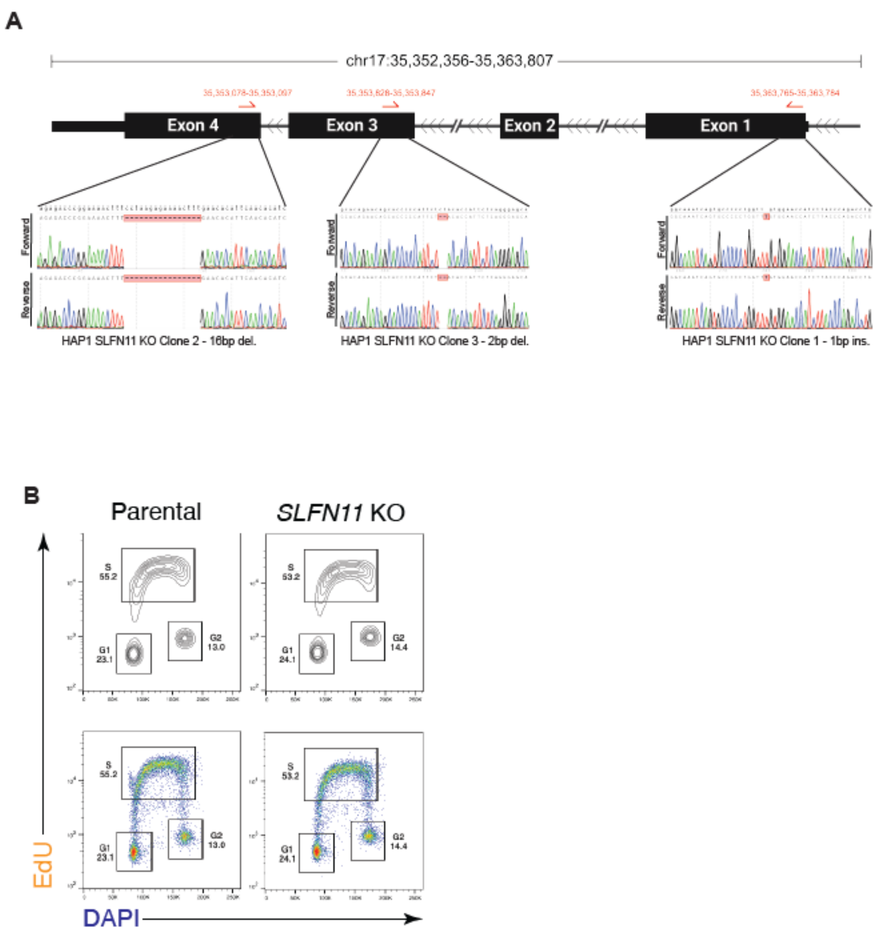

Supplementary Fig. 1.

Supplementary Fig. 1. HAP1 *SLFN11* KO validation and cell cycle analysis. a Genomic coordiates indicating the targeted region for the CRISPR gRNA. Sanger traces of both DNA strands indicates the type of indels induced in each KO clones. **b** Flow cytometry analysis plots depicting the distribution of cell populations at various phases of the cell cycle, comparing between HAP1 parental cells and SLFN11 KO clones

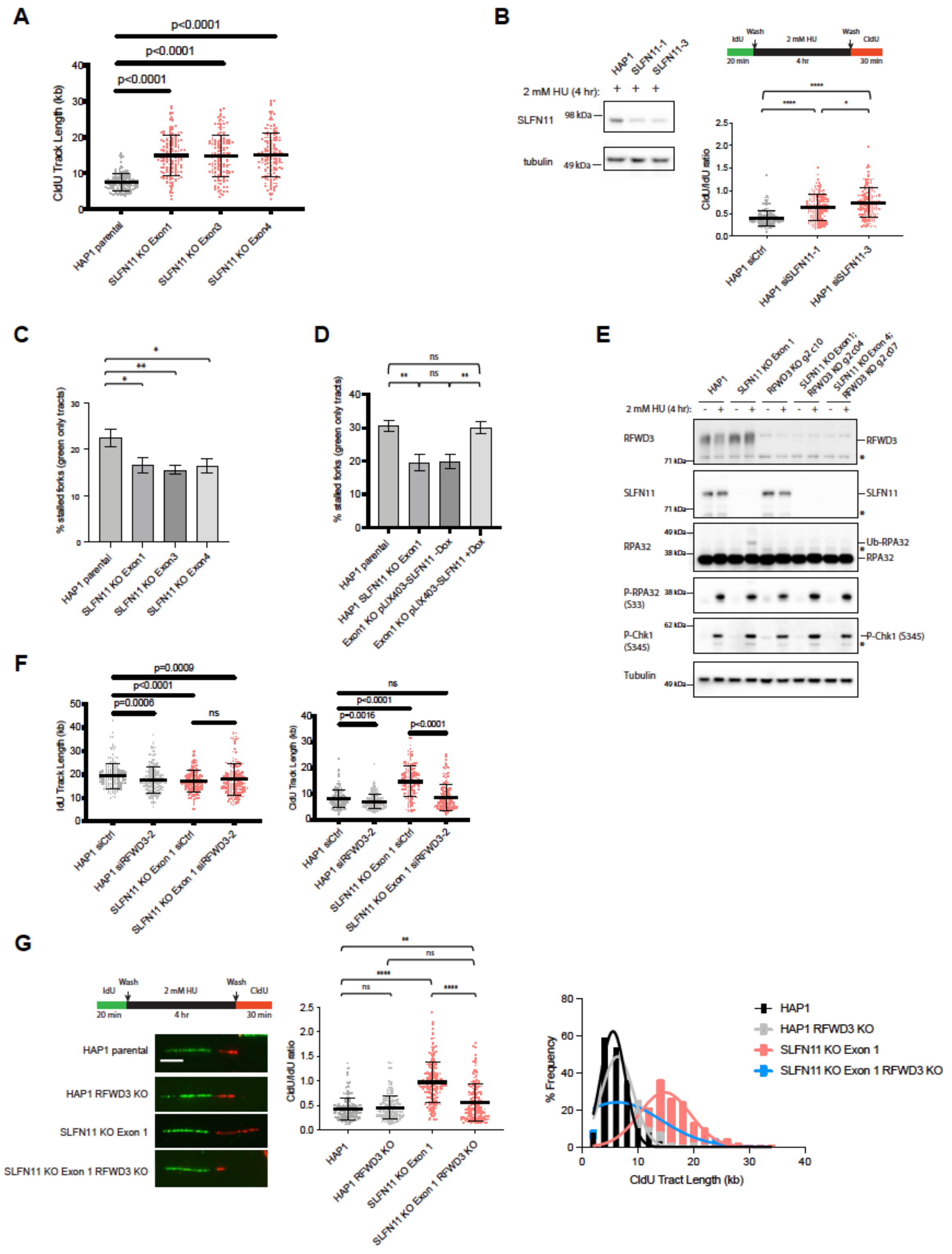

Supplementary Fig. 2.

Supplementary Fig. 2. Validation of CRISPR/Cas9-generated SLFN11/RFWD3 double KO clones. a Swarm plot of CldU tract length measurements from elongating forks in the indicated cells from three independent experiments (n=150 restarted forks) with mean and -/+ SD indicated. p-values were calculated using the Mann-Whitney rank-sum t-test (****p<0.0001, two-tailed). **b** SLFN11 siRNA knockdown causes accelerated fork elongation after replication fork restart. HAP1 cells were treated with or without different SLFN11 siRNAs for 72 hrs and incubated in the presence of 2 mM HU for 4 hrs. Cell lysates were probed with the indicated antibodies for Western blot analysis. Swarm plot showing the ratio of CldU/IdU tract lengths from the replication fork restart assay. Data are plotted for three independent experiments (n=180 restarted forks) with mean and -/+ SD indicated. p-values were calculated using the Mann-Whitney rank-sum t-test (*p<0.05, ****p<0.0001, two-tailed). **c** Quantification of fork restart efficiency (% stalled forks) between parental HAP1 and HAP1 SLFN11 KO cells following recovery after treatment with 2 mM HU for 4 hrs. Data for % stalled forks (left) are represented by green only tracts with the mean -/+ SD of three independent experiments and p-values were calculated using t-test with Welch's correction (*p<0.05, **p<0.01). **d** Quantification of % stalled forks from HAP1 cells treated without or without Dox to induce WT-SLFN11 (right), based on 3 independent experiments. p-values were calculated using t-test with Welch's correction (ns=no significance, **p<0.01). **e** HAP1, SLFN11 KO, RFWD3 KO, SLFN11/RFWD3 double KO cells were treated with or without 2 mM HU for 4 hrs and subjected to Western blot analysis with the indicated antibodies. Asterisks signify non-specific bands. **f** Swarm plot of IdU tract length measurements from elongating forks in the indicated cells from three independent experiments (n=180 elongating forks) with mean and -/+ standard deviation (SD) indicated. p-values were calculated using the Mann-Whitney rank-sum t-test (ns=no significance). Swarm plot of CldU tract length measurements from elongating forks in the indicated cells from three independent experiments (n=180 elongating forks) with mean and -/+ standard deviation (SD) indicated. p-values were calculated using the Mann-Whitney rank-sum t-test (ns=no significance). **g** RFWD3 KO generated in both parental HAP1 and HAP1 SLFN11 KO cells were tested using the replication fork restart assay (see schematic). Representative images of restarted forks from the indicated CRISPR KO cell lines (right). Scatter plot showing the ratio of CldU/IdU tract lengths from the replication fork restart assay. Data are plotted for three independent experiments (n=180 restarted forks) with mean and -/+ SD indicated. p-values were calculated using the Mann-Whitney rank-sum t-test (ns=no significance, **p<0.01, ****p<0.0001, two-tailed). Histogram of CldU tract lengths of restarted elongating forks from the indicated cell lines (right). Scale bar = 5 μM.

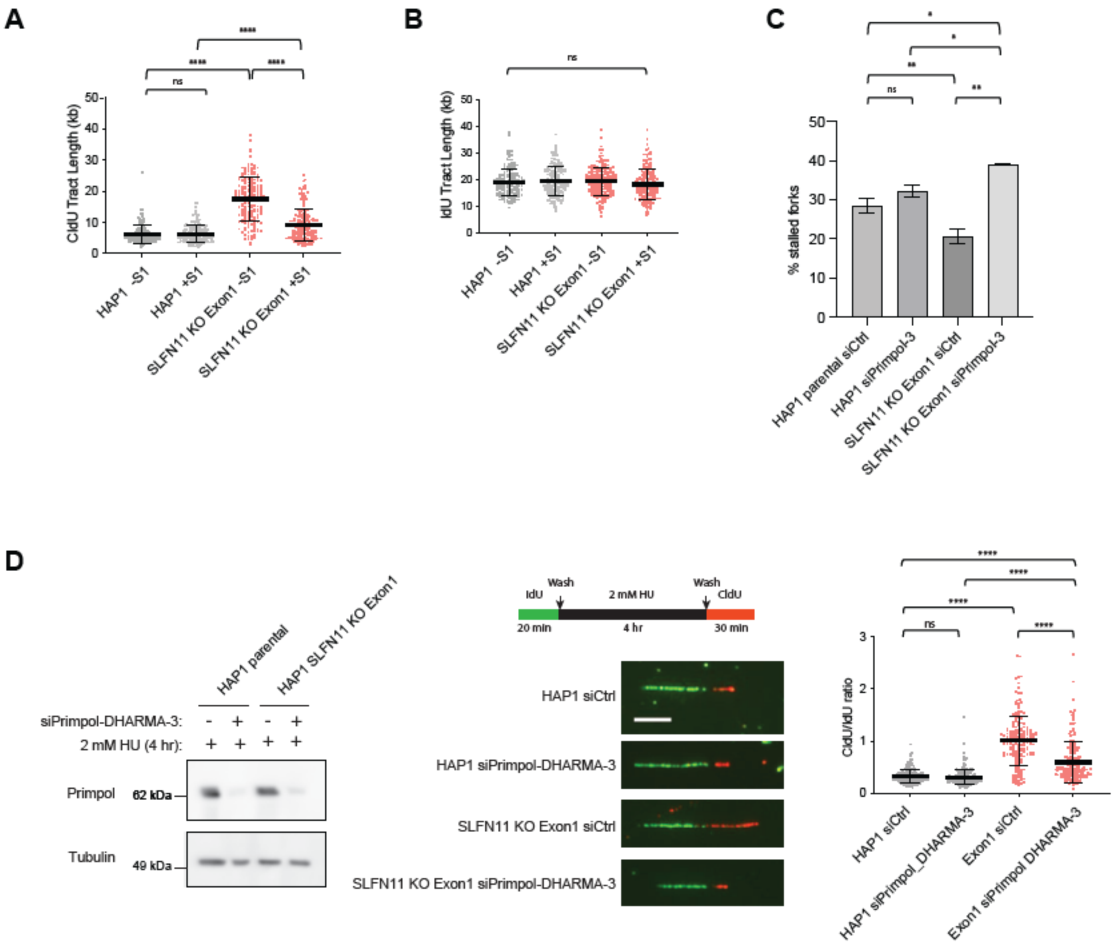

Supplementary Fig. 3.

Supplementary Fig. 3. Effects of SLFN11 and PRIMPOL siRNA knockdown in different cell lines. a Swarm plot and histogram of CldU tract lengths of restarted elongating forks from cells treated with or without S1 nuclease digestion. **b** Swarm plot of IdU tract lengths from the treated samples. **c** Quantification of % stalled forks (green only tracts) following HU treatment with or without Primpol siRNA knockdown based on 3 independent experiments. p-values were calculated using t-test with Welch's correction (ns=no significance, *p<0.05, **p<0.01). **d** PRIMPOL siRNA knockdown reduced DNA synthesis of restarted forks in HAP1 SLFN11 KO cells (using different siRNA sequence, DHARMA-3). HAP1 cells were subjected to Western blot analysis following PRIMPOL siRNA treatment for 72 hours (left). Schematic for replication fork restart assay following knockdown for 72 hours with siRNA against PRIMPOL. Scatter plot shows the ratio of CldU/IdU tract lengths following release from HU, based on three independent experiments (n=180 restarted forks) with mean and -/+ SD indicated (right). p-values were calculated using the Mann-Whitney rank-sum t-test (ns=no significance, ****p<0.0001, two-tailed). Representative images of restarted forks from treated cells (middle). Scale bar = 5 μM.

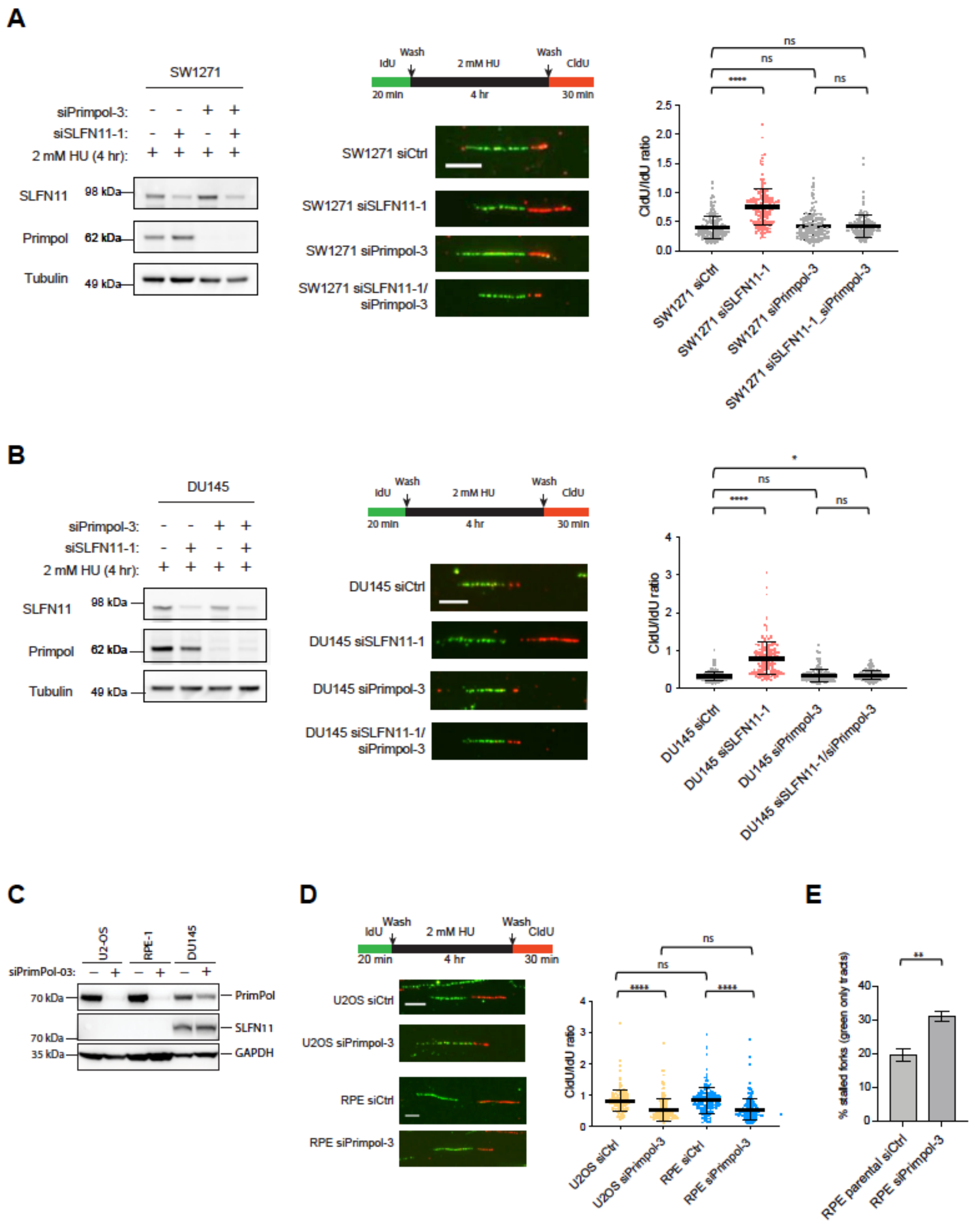

Supplementary Fig. 4.

Supplementary Fig. 4. Effects of SLFN11 and PRIMPOL siRNA knockdown on replication fork restart efficiency are generalizable to other SLFN11-expressing cell lines. a SW1271 cells were treated with siRNAs against either PRIMPOL, SLFN11, or both for 72 hrs and incubated with 2 mM HU for 4 hr. Cell lysates were subjected to Western blot analysis with the indicated antibodies (left). Scatter plot showing the ratio of CldU/IdU tract lengths from the replication fork restart assay (right). Data are plotted for three independent experiments (n=180 restarted forks) with mean and -/+ SD indicated. p-values were calculated using the Mann-Whitney rank-sum t-test (ns=no significance, ****p<0.0001, two-tailed). Representative images of restarted forks from SW1271 cells treated with siRNAs for PRIMPOL and/or SLFN11 (middle). **b** DU145 cells were treated with siRNAs against PRIMPOL, SLFN11, or both for 72 hrs and analyzed for replication fork restart according to schematic. Lysates from cells treated with the indicated siRNAs were subjected to Western blot analysis (left). Scatter plot shows the ratio of CldU/IdU tract lengths following release from HU, based on three independent experiments (n=180 restarted forks) with mean and -/+ SD indicated (right). p-values were calculated using the Mann-Whitney rank-sum t-test (ns=no significance, *p<0.05, ****p<0.0001, two-tailed). Representative images of restarted forks from cells treated as in B (middle). Scale bar = 5 μM. **c** U2OS and RPE-1 cells were treated with or without PRIMPOL siRNA for 72 hrs and incubated with 2 mM HU for 4 hrs. Cell lysates were subjected to Western blot analysis with the indicated antibodies. **d** Representative images of restarted forks from U2OS and RPE cells treated with or without PRIMPOL siRNA. Scatter plot showing the ratio of CldU/IdU tract lengths from the replication fork restart assay (see schematics). Data are plotted for three independent experiments (n=180 restarted forks) with mean and -/+ SD indicated. p-values were calculated using the Mann-Whitney rank-sum t-test (ns=no significance, ****p<0.0001, two-tailed). **e** Quantification of % stalled forks (green only tracts) following treatment with or without siPRIMPOL(right) in RPE-1, based on 3 independent experiments. p-values were calculated using t-test with Welch's correction (**p<0.01). Scale bar = 5 μM.

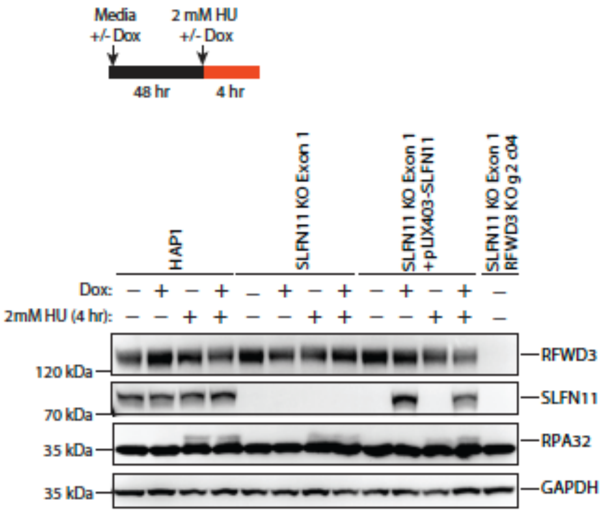

Supplementary Fig. 5.

Supplementary Fig. 5. SLFN11 does not affect RFWD3 protein expression levels. Western blot analysis of HAP1 Parental, SLFN11 KO, SLFN11/RFWD3 double KO, and doxycycline-inducible SLFN11 cells treated with or without 2 mM HU for 4 hours and with or without doxycycline, to assess whether RFWD3 protein levels are affected by SLFN11 expression.

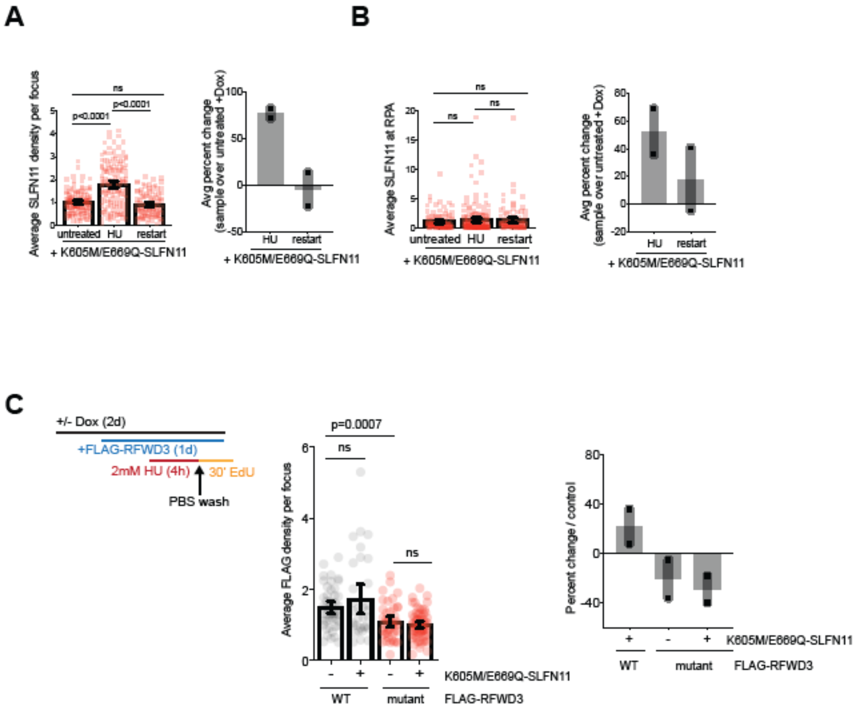

Supplementary Fig. 6.

Supplementary Fig. 6. The ATPase domain of SLFN11 is required for chromatin binding and exclusion of RFWD3 during fork restart. a (Left) Scatterplot quantification measuring the average K605M/E669Q-SLFN11 density per focus in PCNA+ nuclei. *p* values of technical replicates calculated using multiple unpaired two-tailed t-test from two biological replicates (Dox: N=144; Dox, HU: N=147; Dox, restart: N=103). Error bars = mean, SEM. (Right) Bar graph showing percent change of between the average of two biological replicates each normalized to the lowest mean condition relative to no Dox. **b** (Left) Scatterplot quantification measuring the average SLFN11 ATPase mutant density at RPA per focus using pair correlation analysis. *p* values of technical replicates calculated using multiple unpaired two-tailed t-test from two biological replicates (Dox, no HU: N=116; Dox, HU: N=173; Dox, restart: N=103). Error bars = mean, SEM. (Right) Bar graph showing percent change of between the average of two biological replicates each normalized to the lowest mean condition relative to Dox RPEs with no HU treatment. **c** (Left) Scatterplot quantification measuring the average WT- and mutant-RFWD3-FLAG density at PCNA in no dox or dox-induced K605M/E669Q-SLFN11 RPEs. *p* values of technical replicates calculated using multiple unpaired two-tailed t-test from two biological replicates (no Dox+WT: N=48; Dox+WT: N=32; no Dox+mutant: N=45; Dox+mutant: N=66). Error bars = mean, SEM. (Right) Bar graph showing percent change of between the average of two biological replicates each normalized to the lowest mean condition relative to no Dox-WT.

A

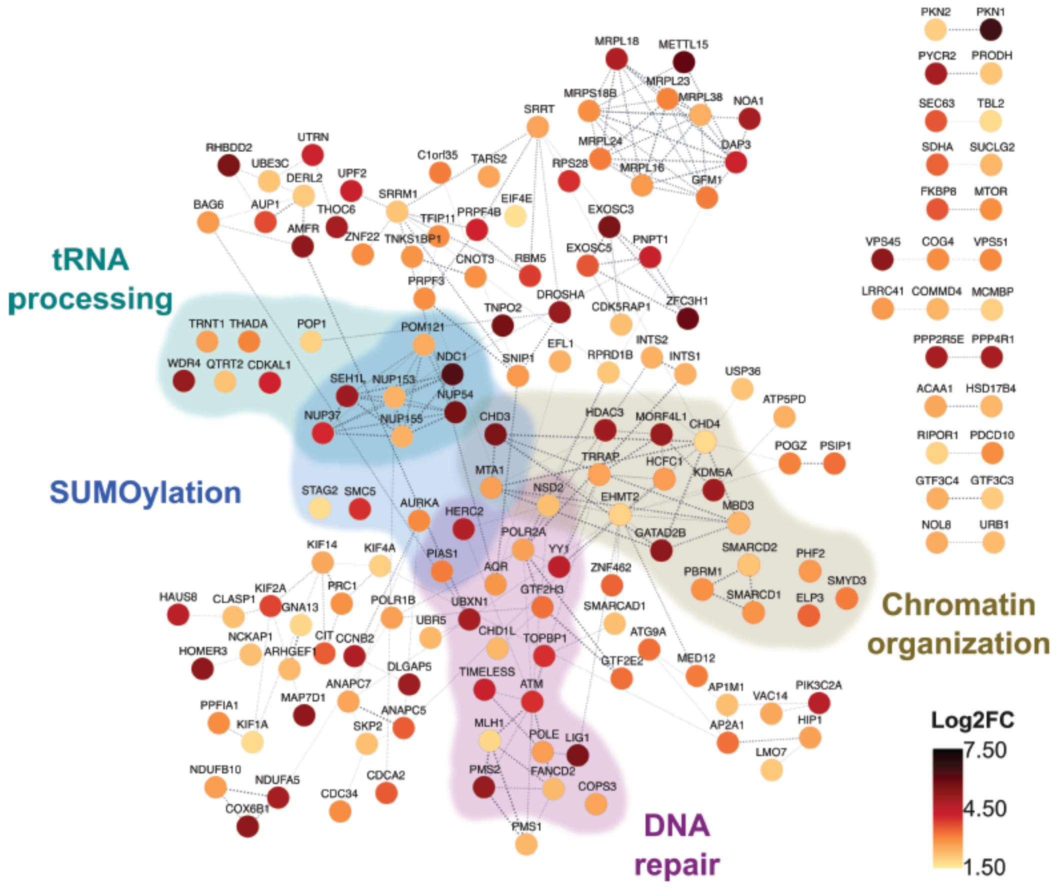

Supplementary Fig. 7.

Supplementary Fig. 7. Cellular pathways revealed by enrichment of RFWD3-interacting proteins in *SLFN11* KO cells under HU-induced replication stress. a STRING network of RFWD3-interacting proteins significantly enriched in SLFN11 KO cells after 4 h treatment with 2 mM HU. The network is drawn based on the STRING physical network (score $\geq$ 0.40). Enriched proteins are grouped into functional Reactome pathways including chromatin organization, DNA repair, SUMOylation, and tRNA processing. Singletons that were not part of the highlighted pathways were removed. Data represent biological triplicates analyzed by SAINT. Figure was created in Cytoscape v3.10.3.

Supplementary Table 1- Key Resources Table

REAGENT or RESOURCE	SOURCE	IDENTIFIER
**Antibodies**		
Mouse monoclonal anti-SLFN11 (E-4)	Santa Cruz Biotechnology	Cat# sc-374339
Mouse monoclonal anti-SLFN11 (D-2)	Santa Cruz Biotechnology	Cat# sc-515071
Rabbit monoclonal anti-GAPDH	Cell Signaling Technology	Cat# 2118L
Rabbit polyclonal anti-CCDC111/PRIMPOL	Proteintech	Cat# 29824-1-AP
Mouse monoclonal anti-FLAG M2	Sigma	Cat# F1804
Rabbit monocloncal anti-FLAG M2	Cell Signaling	Cat# 14793
Mouse monoclonal anti-alpha-Tubulin	Sigma	Cat# CP06
Peroxidase-AffiniPure Goat anti-Rabbit IgG (H+L)	Jackson Labs	Cat# 111-035-003
Peroxidase-AffiniPure Goat anti-Mouse IgG (H+L)	Jackson Labs	Cat# 115-035-003
Mouse monoclonal anti-PCNA	Santa Cruz	Cat# sc-56
Goat anti-Mouse IgG (H+L) AF488 Conjugated	Thermo	Cat# A-11029
Goat anti-Rabbit IgG (H+L) AF647 Conjugated	Thermo	Cat# A-21245
Rabbit polyclonal anti-RFWD3	Novus Biologicals	Cat# NB100-68208
Rabbit monoclonal anti-Chk1 phospho-serine345	Cell Signaling	Cat# 2348S
Rabbit polyclonal anti-RPA32 phospho-serine33	Bethyl	Cat# A300-246A
Rabbit monoclonal anti-RPA32	Bethyl	Cat# A300-244A
Rabbit polyclonal anti-RPA32	Cell Signaling	Cat# 52448S
Rabbit monoclonal anti-RPA32 phospho-serine4/8	Bethyl	Cat# A700-009-T
Rabbit polyclonal anti-RFWD3	Abcam	Cat# ab138030
Mouse monoclonal anti-Chk1	Abcam	Cat# ab69536
Rabbit polyclonal anti-Vinculin	Cell Signaling	Cat# 4650S
**Bacterial and Virus Strains**		
NEB® Stable Competent E. coli (High Efficiency)	New England Biolabs	Cat. # C3040H
**Buffers, Chemicals, Peptides, and Recombinant Proteins**		
Aphidicolin	Cell Signaling Technology	32774S
Camptothecin	Selleck Chemicals	S1288
Ceralasertib (AZD6738)	Selleck Chemicals	S7693
Prexasertib (LY2606368)	Selleck Chemicals	S6385
Cisplatin	Selleck Chemicals	S1166
Paclitaxel	Selleck Chemicals	S1150
Etoposide	Selleck Chemicals	S1225
Bleomycin Sulfate	Selleck Chemicals	S1214
Mitomycin C	Selleck Chemicals	S8146

Temozolomide	Selleck Chemicals	S1237
Talazoparib (BMN 673)	Selleck Chemicals	S7048
Hydroxyurea	ThermoFisher Scientific	A10831.03
Merbarone	Millipore Sigma	M2070
Trypsin-EDTA (0.25%), phenol red	ThermoFisher Scientific/ Invitrogen	25200056
Puromycin Dihydrochloride	ThermoFisher Scientific/ Invitrogen	A1113803
Foundation™ Fetal Bovine Serum	GeminiBio	900-108
Tetracycline Negative FBS	GeminiBio	100-800
S1 Nuclease	ThermoFisher Scientific	EN0321
Benzonase® Nuclease	Sigma	E1014
RIPA Lysis and Extraction Buffer	ThermoFisher Scientific	89901
Halt™ Phosphatase Inhibitor Single-Use Cocktail	ThermoFisher Scientific	78420
Halt™ Protease Inhibitor Cocktail	ThermoFisher Scientific	78437
**Critical Commercial Assays**		
Q5® High-Fidelity 2X Master Mix	New England Biolabs	M0492S
NEBuilder HiFi DNA Assembly Cloning Kit	New England Biolabs	E5520S
CellTiter-Glo® 2.0 Cell Viability Assay	Promega	G9242
GoScript™ Reverse Transcriptase	Promega	A5000
NucleoSpin Gel and PCR Clean-up Columns for gel extraction and PCR clean up	Macherey-Nagel	740609.50S
Gateway™ BP Clonase™ II Enzyme mix	ThermoFisher Scientific/ Invitrogen	11789020
Gateway™ LR Clonase™ II Enzyme mix	ThermoFisher Scientific/ Invitrogen	11791020
Lipofectamine™ 3000 Transfection Reagent	ThermoFisher Scientific/ Invitrogen	L3000001
Neon™ Transfection System 10 µL Kit	ThermoFisher Scientific/ Invitrogen	MPK1096
GelCode™ Blue Safe Protein Stain	ThermoFisher Scientific/ Invitrogen	24594
ZymoPURE II Plasmid Midiprep Kit	Zymo Research	D4201
Anti-FLAG® M2 Magnetic Beads	Sigma	M8823
X-tremeGENE™ HP DNA Transfection Reagent	Roche	6366236001
Click-&-Go Plus EdU 647 Flow Cytometry Assay Kit	Click Chemistry Tools	1381
**Experimental Models: Cell Lines**		
HAP-1	Horizon Discovery	C631
hTERT RPE-1	ATCC	CRL-4000

U2-OS	ATCC	HTB-96
SW1271	ATCC	CRL-2177
DU145	ATCC	HTB-81
HEK293T	ATCC	CRL-3216
**Oligonucleotides**		
SLFN11_Exon1_PCR_For: AGTCTTTTTGAACCATCATCTT	IDT	N/A
SLFN11_Exon1_PCR_Rev: GTCTTTTTGGAAAATTAGGTCAG	IDT	N/A
SLFN11_Exon1_Sanger_For: TGCATGTAACACTTCATTTC	IDT	N/A
SLFN11_Exon1_Sanger_Rev: ATCTACGGTATAATGAAGAA	IDT	N/A
SLFN11_Exon3_PCR_For: CACATGGGTTTAGATGAAGTATACC	IDT	N/A
SLFN11_Exon3_PCR_Rev: CAGAGGCTGGTTTTCACAAA	IDT	N/A
SLFN11_Exon3_Sanger_For: AATAGGGTTATTTCTCTTCC	IDT	N/A
SLFN11_Exon3_Sanger_Rev: AGAGACCTGAAGCCGAGTAA	IDT	N/A
SLFN11_Exon4_PCR_For: TTACCTGGCTCAGGGAAGAC	IDT	N/A
SLFN11_Exon4_PCR_Rev: CTGTCCAACACAATGTGATCACC	IDT	N/A
SLFN11_Exon4_Sanger_For: CTAAGTCACCGTCTTTCTTG	IDT	N/A
SLFN11_Exon4_Sanger_Rev: GTGGGGATGTTAAATGAAGG	IDT	N/A
SLFN11_cDNA_For: ATGGAGGCAAATCAGTGC	IDT	N/A
SLFN11_cDNA_Rev: AGTTTAATAAAGCACATGCAG	IDT	N/A
SLFN11_Exon1_crRNA: GGTTGTGGAACCATCTTACC	IDT	N/A
SLFN11_Exon3_crRNA: CTGAGAATGGTGTAGAGAAT	IDT	N/A
SLFN11_Exon4_crRNA: TGTGTTCAAAGTTTTCTCTT	IDT	N/A
Alt-R® CRISPR-Cas9 tracrRNA	IDT	1072534
**siRNAs**		
siRFWD3-2	Qiagen	5’-ctgagttgtcctgtcacttaa-3’
siSLFN11-1	Qiagen	5’-tcagggaaccttacgaattaa-3’
siSLFN11-3	Qiagen	5’-ttgcaggctctcctaacttaa-3’
siPRIMPOL-3	Qiagen	5’-aagctgtaaagaagacgttca-3’
siPRIMPOL-3-DHARMA	Qiagen	5’-aagaggaaagcuggacaucga-3’

**Recombinant DNA**		
pLIX_403	Addgene	Addgene #41395
pLIX_403_SLFN11	This study	N/A
pLIX_403_SLFN11-E209A/E214A	This study	N/A
pLIX_403_SLFN11-K605M/E669Q	This study	N/A
pLIX_403_SLFN11-K652D	This study	N/A
pSAM504-FLAG-Ub	This study	N/A
psPAX2	Addgene	Addgene #12260
pMD2.G	Addgene	Addgene #12259
Gateway™ pDONR™221 Vector	ThermoFisher Scientific/ Invitrogen	12536017
pDONR221_SLFN11	This study	N/A
pLT3GEPIR-NTC	This study	N/A
pLT3GEPIR-PrimPol-shRNA-C879	This study	N/A
pLT3GEPIR-PrimPol-shRNA-C1257	This study	N/A
pLT3GEPIR-PrimPol-shRNA-C1414	This study	N/A
pLT3GEPIR-PrimPol-shRNA-C1464	This study	N/A
pLT3GEPIR-PrimPol-shRNA-C1490	This study	N/A
pLT3GEPIR-PrimPol-shRNA-C1838	This study	N/A
pLT3GEPIR-RFWD3-shRNA-C1360	This study	N/A
pLT3GEPIR-RFWD3-shRNA-C1780	This study	N/A
pLT3GEPIR-RFWD3-shRNA-C1826	This study	N/A
pLT3GEPIR-RFWD3-shRNA-C4352	This study	N/A
pLT3GEPIR-RFWD3-shRNA-C4917	This study	N/A
FLAG-Human RFWD3-WT, custom codon optimized	This study	N/A
FLAG-Human RFWD3-mutant, custom codon optimized	This study	N/A
**Software and Algorithms**		
GraphPad Prism	GraphPad Software Inc	http://www.graphpad.com/scientific-software/prism/
Flow Jo 10 software	Flowjo, LLC	https://www.flowjo.com/
MatLab (v2017b)	https://www.mathworks.com/	MatLab (v2017b)
R Statistical Software v4.4.2	The R Foundation	https://www.r-project.org
clusterProfiler v4.14.4	Bioconductor v3.20	https://bioconductor.org/packages/release/bioc/html/clusterProfiler.html
Cytoscape v3.10.3	The Cytoscape Consortium	https://cytoscape.org
stringApp v2.2.0	Doncheva et al. (PMID: 30450911)	https://apps.cytoscape.org/apps/stringApp
