# Supplementary material for: SLFN11 counteracts the RFWD3-PRIMPOL DNA damage tolerance axis to restrain gapped DNA synthesis in response to replication stress
Source: Nat Commun. 2025 Dec 10;16:11029. doi: 10.1038/s41467-025-66068-1 (PMC12696100; doi:10.1038/s41467-025-66068-1)
Supplement: Supplementary file 2 — Reporting Summary [file 41467_2025_66068_MOESM2_ESM.pdf]

nature portfolio

Corresponding author(s): Tony T Huang and John T Poirier

Last updated by author(s): Jul 22, 2025

Reporting Summary

Nature Portfolio wishes to improve the reproducibility of the work that we publish. This form provides structure for consistency and transparency in reporting. For further information on Nature Portfolio policies, see our Editorial Policies and the Editorial Policy Checklist.

Statistics

For all statistical analyses, confirm that the following items are present in the figure legend, table legend, main text, or Methods section.

n/a	Confirmed	
☐	☒	The exact sample size (*n*) for each experimental group/condition, given as a discrete number and unit of measurement
☐	☒	A statement on whether measurements were taken from distinct samples or whether the same sample was measured repeatedly
☐	☒	The statistical test(s) used AND whether they are one- or two-sided<br>*Only common tests should be described solely by name; describe more complex techniques in the Methods section.*
☒	☐	A description of all covariates tested
☐	☒	A description of any assumptions or corrections, such as tests of normality and adjustment for multiple comparisons
☐	☒	A full description of the statistical parameters including central tendency (e.g. means) or other basic estimates (e.g. regression coefficient) AND variation (e.g. standard deviation) or associated estimates of uncertainty (e.g. confidence intervals)
☐	☒	For null hypothesis testing, the test statistic (e.g. *F*, *t*, *r*) with confidence intervals, effect sizes, degrees of freedom and *P* value noted<br>*Give P values as exact values whenever suitable.*
☒	☐	For Bayesian analysis, information on the choice of priors and Markov chain Monte Carlo settings
☒	☐	For hierarchical and complex designs, identification of the appropriate level for tests and full reporting of outcomes
☒	☐	Estimates of effect sizes (e.g. Cohen's *d*, Pearson's *r*), indicating how they were calculated

Our web collection on statistics for biologists contains articles on many of the points above.

Software and code

Policy information about availability of computer code

Data collection	Super-resolution microscopy data were acquired using Micro-Manager (vl.4)<br>Please see Methods section for detailed data acquisition description
Data analysis	Super-resolution image reconstruction was performed through C++ (via Intel Core i7 7800X) and CUDA8.0 (via NVIDIA GTX 1060) using the Maximum Likelihood Estimation (MLE) algorithm.<br>Codes for Pair-Correlation algorithms, as well as a testing demo (with simulation codes) are available at https://github.com/yiny02/direct-Triple-Correlation-Algorithm. The code is for Research and Educational Purposes for Non-Profit Academic and/or Research Institutions.<br>Data analysis software includes ImageJ 1.52a, GraphPad Prism (v8), and Matlab (v2017b)<br>Data presentation/graphing were performed through Matlab (v2017b), GraphPad Prism (v8)

For manuscripts utilizing custom algorithms or software that are central to the research but not yet described in published literature, software must be made available to editors and reviewers. We strongly encourage code deposition in a community repository (e.g. GitHub). See the Nature Portfolio guidelines for submitting code & software for further information.

Data

Policy information about availability of data

All manuscripts must include a data availability statement. This statement should provide the following information, where applicable:

- Accession codes, unique identifiers, or web links for publicly available datasets
- A description of any restrictions on data availability
- For clinical datasets or third party data, please ensure that the statement adheres to our policy

All imaging and single-molecule data constitute a sizable dataset (>l0TB) that cannot be reasonably maintained online. Raw data will be made available by the corresponding author upon request.

Research involving human participants, their data, or biological material

Policy information about studies with human participants or human data. See also policy information about sex, gender (identity/presentation), and sexual orientation and race, ethnicity and racism.

Reporting on sex and gender	N/A
Reporting on race, ethnicity, or other socially relevant groupings	N/A
Population characteristics	N/A
Recruitment	N/A
Ethics oversight	N/A

Note that full information on the approval of the study protocol must also be provided in the manuscript.

Field-specific reporting

Please select the one below that is the best fit for your research. If you are not sure, read the appropriate sections before making your selection.

☒ Life sciences ☐ Behavioural & social sciences ☐ Ecological, evolutionary & environmental sciences

For a reference copy of the document with all sections, see nature.com/documents/nr-reporting-summary-flat.pdf

Life sciences study design

All studies must disclose on these points even when the disclosure is negative.

Sample size	For all experiments, sample size was not predetermined, and as much data as possible was collected depending on the nature of the experiments or in order to perform proper statistical analysis.
Data exclusions	For all super-resolution imaging experiments, PCNA(+) S-phase nuclei were selected for analysis, which were then analyzed equally. No other data exclusion was performed
Replication	All super-resolution experiments were performed at least in duplicate with >60 sample size, as indicated in the manuscript text.<br>Western blotting experiments were performed in at least three independent experiments.<br>DNA fibers analysis was performed in at least three independent experiments.<br>We followed the same protocols to generate replicates for each of our experiments, and the analysis of the data was reliably reproduced.
Randomization	For all imaging experiments, DNA fibers on coverslips were randomly selected for imaging.<br>For all super-resolution experiments, PCNA+ regions of interest were randomly selected for imaging.
Blinding	For all data and imaging experiments, blinding was not possible as experimental conditions were evident from the imaging data. Image processing and analysis were done using computational pipelines that were applied equally to all conditions and replicates, therefore do not require blinding.

Reporting for specific materials, systems and methods

We require information from authors about some types of materials, experimental systems and methods used in many studies. Here, indicate whether each material, system or method listed is relevant to your study. If you are not sure if a list item applies to your research, read the appropriate section before selecting a response.

Materials & experimental systems

n/a	Involved in the study
☐	☒ Antibodies
☐	☒ Eukaryotic cell lines
☒	☐ Palaeontology and archaeology
☒	☐ Animals and other organisms
☒	☐ Clinical data
☒	☐ Dual use research of concern
☒	☐ Plants

Methods

n/a	Involved in the study
☒	☐ ChIP-seq
☐	☒ Flow cytometry
☒	☐ MRI-based neuroimaging

Antibodies

Antibodies used

Mouse monoclonal anti-SLFN11 (E-4) Santa Cruz Biotechnology Cat# sc-374339
Mouse monoclonal anti-SLFN11 (D-2) Santa Cruz Biotechnology Cat# sc-515071
Rabbit monoclonal anti-GAPDH Cell Signaling Technology Cat# 2118L
Rabbit polyclonal anti-CCDC111/PRIMPOL Proteintech Cat# 29824-1-AP
Mouse monoclonal anti-FLAG M2 Sigma Cat# F1804
Rabbit monocloncal anti-FLAG M2 Cell Signaling Cat# 14793
Mouse monoclonal anti-alpha-Tubulin Sigma Cat# CP06
Peroxidase-AffiniPure Goat anti-Rabbit IgG (H+L) Jackson Labs Cat# 111-035-003
Peroxidase-AffiniPure Goat anti-Mouse IgG (H+L) Jackson Labs Cat# 115-035-003
Mouse monoclonal anti-PCNA Santa Cruz Cat# sc-56
Goat anti-Mouse IgG (H+L) AF488 Conjugated Thermo Cat# A-11029
Goat anti-Rabbit IgG (H+L) AF647 Conjugated Thermo Cat# A-21245
Rabbit polyclonal anti-RFWD3 Novus Biologicals Cat# NB100-68208
Rabbit monoclonal anti-Chk1 phospho-serine345 Cell Signaling Cat# 2348S
Rabbit polyclonal anti-RPA32 phospho-serine33 Bethyl Cat# A300-246A
Rabbit monoclonal anti-RPA32 Bethyl Cat# A300-244A
Rabbit polyclonal anti-RPA32 Cell Signaling Cat# 52448S
Rabbit monoclonal anti-RPA32 phospho-serine4/8 Bethyl Cat# A700-009-T
Rabbit polyclonal anti-RFWD3 Abcam Cat# ab138030
Mouse monoclonal anti-Chk1 Abcam Cat# ab69536
Rabbit polyclonal anti-Vinculin Cell Signaling Cat# 4650S

Validation

https://www.scbt.com/p/slfn11-antibody-e-4?srsltid=AfmBOopstlfskEw10DSCOMymbHI1nEylDdHK31ZFKmqrCchAwiAIxrb4
https://www.scbt.com/p/slfn11-antibody-d-2?srsltid=AfmBOoqiSsNLErQrqSP5HrDmss6j4iBju1AdMd7yLhtPiCDhNu8emeiY
https://www.cellsignal.com/products/primary-antibodies/gapdh-14c10-rabbit-mab/2118?srsltid=AfmBOoqAfioKFjjk-OJtXgwQBMd5zQrFRBK8ekB2t3jvewVDWjOXDJ9Z
https://www.ptglab.com/products/CCDC111-Antibody-29824-1-AP.htm?srsltid=AfmBOoqSuZ3ZFnfgJLWAkcDfh_WGN6YVb7nG0zcnsr7q5DgMyyRKsniF
https://www.sigmaaldrich.com/US/en/product/sigma/f3165?srsltid=AfmBOoqMmmyb0JW_CfD5N7Or33vlNp4O5sCh7az4zafk2WKn1BURi7nT
https://www.cellsignal.com/products/primary-antibodies/dykddddk-tag-d6w5b-rabbit-mab-binds-to-same-epitope-as-sigma-aldrich-anti-flag-m2-antibody/14793?srsltid=AfmBOopGpmSX3LPTcgIXEdUht_Q5T6e8tg3xQ6IOJkm4C6x6doweFv2a
https://www.sigmaaldrich.com/US/en/product/mm/cp06?srsltid=AfmBOoodEMrlHbpTtKLUpnB69spHJYDzDTu082dtfKojPyI3notU1CE5
https://www.jacksonimmuno.com/catalog/products/111-035-003
hhttps://www.jacksonimmuno.com/catalog/products/115-035-044
https://www.scbt.com/p/pcna-antibody-pc10?srsltid=AfmBOoqQcXBpXVg_-VT6UWEtAj5xPBTKt05zpAEKRqnWNJd3ayLp_Op3
https://www.thermofisher.com/antibody/product/Goat-anti-Mouse-IgG-H-L-Cross-Adsorbed-Secondary-Antibody-Polyclonal/A-11001
https://www.thermofisher.com/antibody/product/Goat-anti-Rabbit-IgG-H-L-Cross-Adsorbed-Secondary-Antibody-Polyclonal/A-21244
https://www.novusbio.com/products/rfwd3-antibody_nb100-68208?srsltid=AfmBOoqMRQf1QPthiEBBgL8U2fF1BqV5uZLCuWDVEz9-TDF4v2F__DFv
https://www.cellsignal.com/products/primary-antibodies/phospho-chk1-ser345-133d3-rabbit-mab/2348?srsltid=AfmBOopDCxJuLe8u1MqZBdkga3s61SVrYJHh-9O6o0heEgMq0XOxHLiX
https://www.fortislife.com/products/primary-antibodies/rabbit-anti-phospho-rpa32-s33-antibody/BETHYL-A300-246
https://www.cellsignal.com/products/primary-antibodies/rpa32-rpa2-antibody/52448?srsltid=AfmBOopJozrtKLI066liB548Zqx3kWntaXIUJdAUF-nlvtoA9elqgFUC
https://www.fortislife.com/products/primary-antibodies/rabbit-anti-phospho-rpa32-s4-s8-recombinant-monoclonal-antibody-bl-165-5f1/BETHYL-A700-009
https://www.abcam.com/en-us/products/primary-antibodies/rfwd3-antibody-ab138030?srsltid=AfmBOoqJEtCpJQ-czgljJ7pHRm79W03nxL4BGhv0ctX-FOXgFzU2N3YQ
https://www.cellsignal.com/products/primary-antibodies/vinculin-antibody/4650?srsltid=AfmBOor-UAvvXjhm_32YZL2tPG9WnDr8r4XBwVbEmTgNyoaQ8d1cWGDI

Eukaryotic cell lines

Policy information about cell lines and Sex and Gender in Research

Cell line source(s)	All cell lines were obtained from ATCC
Authentication	no cell line authentication was performed
Mycoplasma contamination	Cell lines were tested for mycoplasma using Universal Mycoplasma Detection Kit (ATCC), all cell lines used tested negative for mycoplasma
Commonly misidentified lines (See ICLAC register)	no commonly misidentified lines were used

Plants

Seed stocks	N/A
Novel plant genotypes	N/A
Authentication	N/A

Flow Cytometry

Plots

Confirm that:

☒ The axis labels state the marker and fluorochrome used (e.g. CD4-FITC).

☒ The axis scales are clearly visible. Include numbers along axes only for bottom left plot of group (a 'group' is an analysis of identical markers).

☒ All plots are contour plots with outliers or pseudocolor plots.

☒ A numerical value for number of cells or percentage (with statistics) is provided.

Methodology

Sample preparation	Cell cycle analysis was performed using the Invitrogen Click-iT™ EdU Alexa Fluor™ 488 Flow Cytometry Assay Kit according to manufacturer's instructions. Briefly, asynchronous, actively replicating adherent cells were pulsed with EdU nucleotide analog, washed with PBS, harvested, fixed, and permeabilized. A flurophores were ligated to EdU molecules using click chemistry. DNA content was labeled with DAPI and cells were analyzed by FACS
Instrument	BD FACS Symphony
Software	FLowJo
Cell population abundance	at least 10,000 cells per sample
Gating strategy	negative controls (no EdU, no DAPi, no EdU nor DAPI) were used for gating. doublets and cell aggregates were excluded in gating

☒ Tick this box to confirm that a figure exemplifying the gating strategy is provided in the Supplementary Information.
